# Effects of nanosilver and nanozinc incorporated mesoporous calcium-silicate nanoparticles on the mechanical properties of dentin

**DOI:** 10.1371/journal.pone.0182583

**Published:** 2017-08-07

**Authors:** Jie Zhu, Ruizhen Liang, Chao Sun, Lizhe Xie, Juan Wang, Diya Leng, Daming Wu, Weihong Liu

**Affiliations:** 1 Jiangsu Key Laboratory of Oral Diseases, Nanjing Medical University, Nanjing, China; 2 Department of Endodontics, Affiliated Hospital of Stomatology, Nanjing Medical University, Nanjing, China; 3 Department of Radiology, Affiliated Hospital of Stomatology, Nanjing Medical University, Nanjing, China; Institute of Materials Science, GERMANY

## Abstract

Mesoporous calcium-silicate nanoparticles (MCSNs) are advanced biomaterials for drug delivery and mineralization induction. They can load silver and exhibit significantly antibacterial effects. However, the effects of MCSNs and silver-loaded MCSNs on dentin are unknown. The silver (Ag) and/or zinc (Zn) incorporated MCSNs (Ag-Zn-MCSNs) were prepared by a template method, and their characterizations were tested. Then the nanoparticles were filled into root canals and their effects on the dentin were investigated. Ag-Zn-MCSNs showed characteristics of mesoporous materials and sustained release of ions over time. Ag-Zn-MCSNs adhered well to the root canal walls and infiltrated into the dentinal tubules after ultrasound activation. Ag-Zn-MCSNs showed no significantly negative effects on either the flexural strength or the modulus of elasticity of dentin, while CH decreased the flexural strength of dentin significantly (*P*<0.05). These findings suggested that Ag and Zn can be incorporated into MCSNs using a template method, and the Ag-Zn-MCSNs may be developed into a new disinfectant for the root canal and dentinal tubules.

## Introduction

The essential elements for successful outcomes of endodontic treatment are eliminating or significantly reducing bacterial biofilms and preventing recontamination of the root canal system after treatment [1]. However, bacteria biofilm structures in infected root canals, the anatomical complexities of the root canal system, dentin structure/composition, and the limitations associated with chemical disinfectants present challenges for endodontic disinfection [1, 2].

One necessary element in the control of endodontic infection is the use of local intracanal medicaments between appointments. Calcium hydroxide (CH) has been widely used as a routine antimicrobial intracanal medication in endodontics [3]. The antibacterial property of CH is related to the release of hydroxyl ions in an aqueous environment. Hydroxy ions are highly oxidant free radicals that show extreme reactivity; they react with several biomolecules, including damage to the bacterial cytoplasmic membrane, protein denaturation, and damage to the DNA [3, 4]. However, the antibacterial potential and tissue dissolution capacity of CH is decreased by dentin, hydroxylapatite and remnants of necrotic pulp tissue [5, 6]. The low solubility and diffusibility of CH may make it difficult for CH to produce a rapid and significant increase in the pH to eliminate bacteria located within dentinal tubules and anatomical variations [7]. Moreover, the use of CH dressing for an extended period may reduce the flexural strength and lower the fracture resistance of dentin, thus increasing the risk of tooth fracture [8, 9] and resulting in the failure of root canal treatment (RCT).

Zinc (Zn) is an important mineral that is required for normal bone development. It has biphasic effects on the differentiation and mineralization of human osteoblast-like cells [10]. Another important property of Zn is that it possesses broad spectrum bactericidal effects and has been used in medicines for infection control [11], just like silver (Ag). Studies have indicated that Zinc oxide nanoparticles (ZnO-NPs) can deform and damage *Pseudomonas aeruginosa*, *Staphylococcus aureus* and *E*.*coli*, and can significantly inhibit the growth of bacteria[12, 13]. ZnO-NPs also significantly inhibit bacterial adherence to dentin, which would prevent bacterial recolonization and biofilm formation on dentin[14].

Recently, the development of bactericides and disinfectants prepared by loading metal ions onto inorganic carriers has received extensive attention. Zn^2+^-containing silicate-based bioceramics have shown improved chemical stability and mechanical properties compared with CaSiO_3_ bioceramics [15]. Mg^2+^- and Zn^2+^-containing silicate materials showed desirable features for promoting cell proliferation, osteogenic differentiation and bacteria suppression [16]. Zn-Ag-loaded nano-SiO_2_ did not change the structure of the nano-SiO_2_ but exhibited excellent antibacterial properties against *E*. *coli* and *S*. *Faecalis* [17].

Mesoporous calcium-silicate nanoparticles (MCSNs) are advanced biomaterials because they possess excellent apatite-mineralization formation properties in the apical root canal of teeth and promote the osteogenic differentiation of stem cells in vitro [18]. They have highly ordered mesopore channel structures and possess a more optimal surface area and pore volume, which makes them excellent platforms for the efficient delivery of drugs, such as antibacterial reagents and antibiotics [18, 19]. Our previous studies indicated that Ag-incorporated MCSNs (Ag-MCSNs) prepared using both the adsorption and template methods showed the characteristic morphology of mesoporous materials, low cytotoxicity and significant antibacterial effects [20]. However, there are few studies of Zn-incorporated MCSNs (Zn-MCSNs) and Ag-Zn-MCSNs at present, and the effects of such MCSNs on the mechanical properties of dentin are unknown. Therefore, the purpose of this study was to prepare Zn-MCSNs and Ag-Zn-MCSNs and to investigate their effects on dentin.

## Materials and methods

### Synthesis and characterizations

MCSNs were synthesized using a template method according to Wu *et al* [18]. Briefly, 6.6 g of cetyltrimethylammonium bromide (CTAB, Sigma-Aldrich, St Louis, MO, USA) and 12 mL of ammonium hydroxide were dissolved in 600 mL of deionized water by stirring for 30 minutes. Then, 30 mL of tetraethyl orthosilicate (TEOS, Sinopharm Chemical Reagent Co., Ltd., China) and 31.21 g of calcium nitrate tetrahydrate (Kelong of Chengdu Chemical Reagent Co., Ltd., China) were added and stirred vigorously for 3 hours. The products were collected by filtration and washed three times each with deionized water and ethanol. Then, the collected powders were dried at 60°C overnight and calcined at 550°C for 2 hours to remove remaining traces of CTAB.

The Ag-MCSNs, Zn-MCSNs and Ag-Zn-MCSNs were also synthesized according to our previously described method [20]. The same procedures were employed except that silver nitrate and/or zinc nitrate (Reagent No.1 Factory of Shanghai Chemical Reagent Co., Ltd, China) (Table 1) were added together with 30 mL of TEOS and 31.21 g of calcium nitrate tetrahydrate.

**Table 1:** The molar ratio of Ag/Zn and the weight of silver nitrate and zinc nitrate added in the synthesis of the nanoparticles.

Nanoparticles	Ag: Zn (molar)	Silver nitrate	Zinc nitrate
Ag-MCSNs	-	4.911 g	-
Zn-MCSNs	-	-	8.580 g
Ag-Zn-MCSNs	1: 1	2.456 g	4.290 g

The prepared MCSNs, Ag-MCSNs, Zn-MCSNs and Ag-Zn-MCSN+s were characterized using field emission-scanning electron microscopy (FE-SEM, 1530 VP; LEO, Germany), transmission electron microscopy (TEM, JEM-2100; JEOL, Tokyo, Japan), energy dispersive spectrometry (EDS, I MCA 300; OXFORD, UK). Brunauer–Emmett–Teller (BET) and Barrett–Joyner–Halenda (BJH) analyses were used to determine the specific surface area, pore volume, and pore size distribution according to N2 adsorption–desorption isotherms (ASAP 2020; Micromeritics, Norcross, GA, USA).

### Ions release and pH measurement

Twenty milligrams of MCSNs, Ag-MCSNs, Zn-MCSNs and Ag-Zn-MCSNs were soaked in 10 mL of deionized water at room temperature. At 9 days, the solution was adsorbed to measure the released Ca^2+^, Si^4+^, Ag^+^ and Zn^2+^ concentrations using inductively coupled plasma-atomic emission spectrometry (ICP-AES, Prodigy 7; Leeman, USA). For the pH measurement of MCSNs, Ag-MCSNs, Zn-MCSNs, Ag-Zn-MCSNs and CH, 150 mg of the material was soaked in 30 mL deionized water at 37°C. The pH change over time was measured using a pH meter (SIN-PH100, Sinomeasure, China) in 14 days.

### Adhesion and infiltration test on dentin

Human single-rooted mandibular premolars with mature apices were collected between December 2015 and February 2016 from the Department of Oral and Maxillofacial Surgery under a protocol approved by the Ethics Committee of the Affiliated Hospital of Stomatology, Nanjing Medical University (PJ2015-038-001). All clinical investigation have been conducted according to the principles expressed in the Declaration of Helsinki, and the written informed consent have been obtained from the participants. The crowns of the teeth were removed, and the root lengths were standardized to 12 mm. Working length was established at 1 mm short of the anatomical apex. The canals were instrumented with ProTaper nickel titanium rotary instruments (X-SmartTM, Dentsply Maillefer, Japan) to size F3. The canals were irrigated using a 27-gauge needle and 2 mL of 2% sodium hypochlorite (NaOCl) when changing files. Then, 6 mL of 5.25% NaOCl and 6 mL of 17% ethylenediaminetetraacetic acid was used as the final irrigant to completely remove dentinal debris from the canal wall. Finally, the instrumented canals were rinsed with a large amount of water to remove the residual irrigant.

The instrumented roots were divided randomly into three groups. In group 1, the roots were soaked in saline. In group 2, the root canals of the teeth were filled with CH suspension (CH: saline = 1: 1.5) using a size 15 stainless steel K file. In group 3, Ag-Zn-MCSNs suspension (Ag-Zn-MCSNs: saline = 1: 3) was prepared and injected into the canals using a pipettor. The suspension in the root canals was subjected to passive ultrasonic vibration by inserting an ultrasonic needle into the canal to a depth 1 mm short of the working length. The power of the ultrasonic device (P5XS, Satelec, Cedex, France) was set at scale 4, and two 30-second sessions of ultrasonic vibration were applied. All the roots were stored at 37°C and 100% humidity.

After 7 days, the CH and Ag-Zn-MCSNs suspensions in the root canals were removed with 5 mL of saline. Then, the roots were stored at 60°C for 12 hours. The roots were then split into two halves longitudinally in the buccolingual direction with a hammer and chisel to expose the root canal wall and axial cross-sections of the dentinal tubules. FE-SEM was used to observe the adhesion of Ag-Zn-MCSNs to the dentin surface and the infiltration of nanoparticles into the dentinal tubules. Mapping of the elemental distribution was performed for the canal wall dentin to confirm the adhesion of Ag-Zn-MCSNs to the dentin surfaces and the distribution of the Ag and Zn elements. EDS analyses were performed on the nanoparticles to confirm the infiltration of Ag-Zn-MCSNs.

### Effects on the mechanical properties of root dentin

Forty-eight single-rooted mandibular premolars were prepared as described above and divided into 6 groups randomly. The roots were split into two halves: one half of the roots in all groups were horizontally sectioned into 1-mm-wide specimens from the cementoenamel junction (CEJ) to the apex with a low-speed saw (Isomet; Buehler Ltd, USA). Two or three sequential 1-mm-wide specimens were obtained from each half of the root. The cementum was removed from the root surface and the dentin strip was prepared to the size of 1×1×3 mm (width × thickness × length). During the preparation, the surface of the root canal was not touched. The specimens were then subjected to mechanical testing. Three-point bending tests were performed using a universal testing machine (R3365; INSTRON, USA) to test the mechanical properties of the root dentin. For the testing apparatus, a specimen holder with two cylindrical supports with a span of 1 mm was used. The cross-head speed of the testing machine was set to 0.5 mm/min, and the specimens were tested until they fractured. The test machine software automatically recorded the load at fracture and the modulus of elasticity. Flexural strength was calculated using the formula 3*PL*/*2bd*^*2*^, where *P* = load at fracture, *L* = length of support span, *b* = strip width, and *d* = strip thickness.

The other half of the roots in group 1 were soaked in saline as the control, and the other half of the root canals in group 2–6 were filled with a suspension (CH, MCSNs, Ag-MCSNs, Zn-MCSNs and Ag-Zn-MCSNs, respectively) prepared as described above. All the roots were stored at 37°C and 100% humidity. After 30 days, the canals were rinsed with 5 mL of saline to remove the suspension. They were then prepared and the mechanical properties of the root dentin were tested in the manners described above.

### Statistical analysis

Statistical analyses were performed using SPSS19.0 software (IBM Corp., Armonk, NY). All the data were expressed as the means ± standard deviation (SD). The control and test groups were compared using the paired-samples T-test. Statistical significance was preset at *P* = 0.05.

## Results

### Characterizations

**Fig 1:**
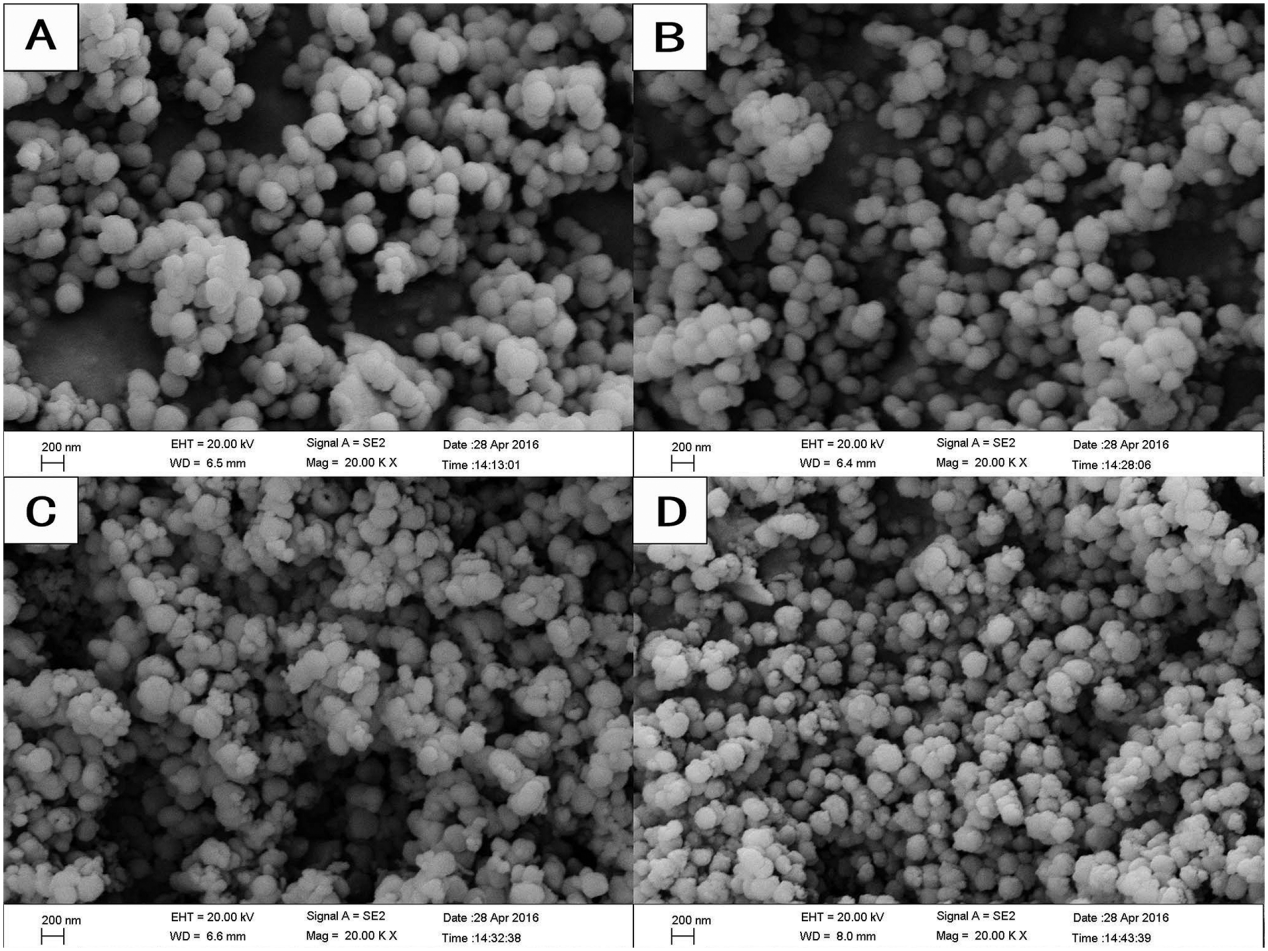
FE-SEM images of the nanoparticles. (A)MCSNs, (B)Ag-MCSNs, (C)Zn-MCSNs, (D)Ag-Zn-MCSNs.

**Fig 2:**
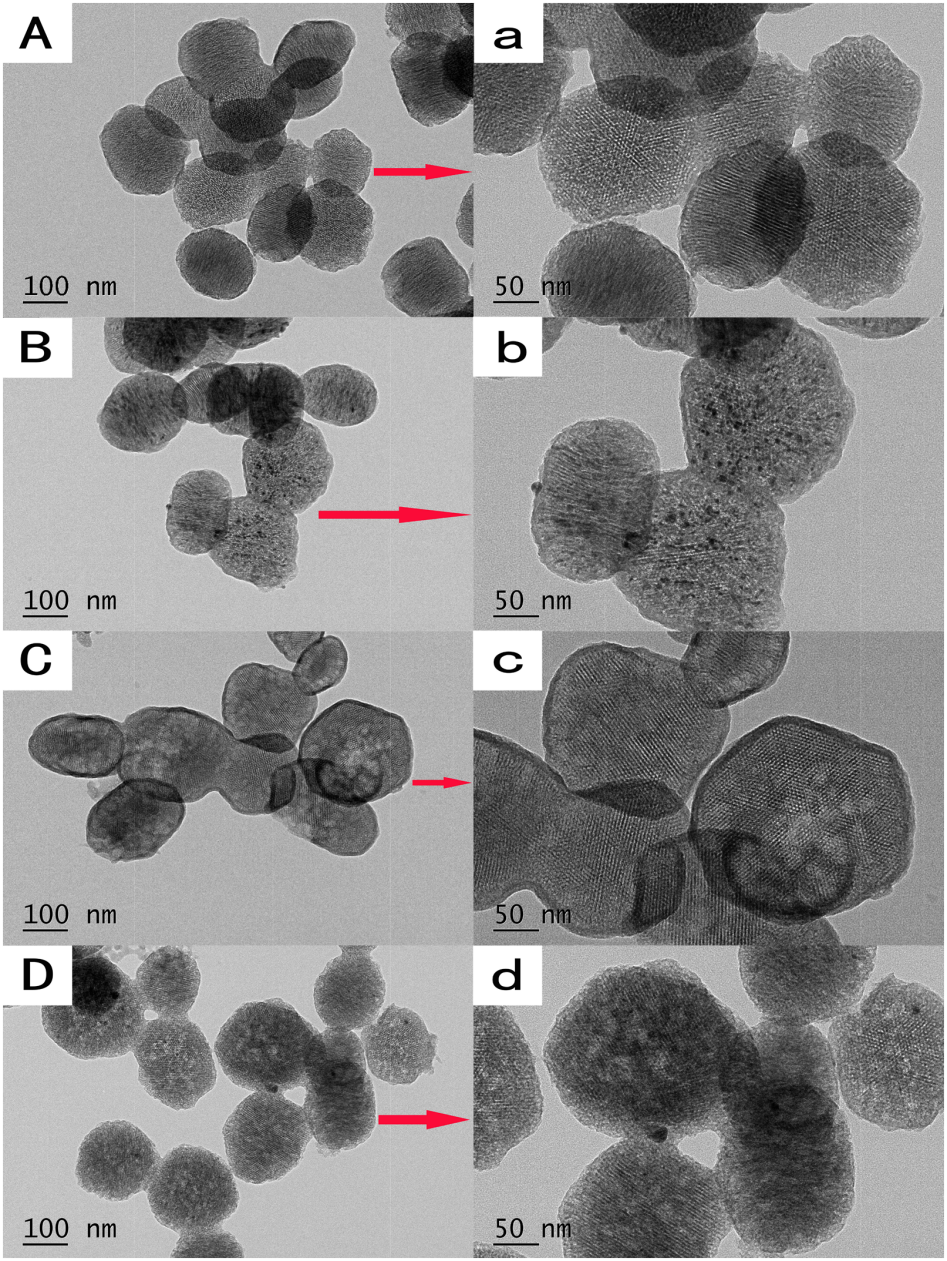
TEM images of the nanoparticles. (A)MCSNs, (B)Ag-MCSNs, (C)Zn-MCSNs, (D)Ag-Zn-MCSNs. Magnified images (a-d) of the selected area (arrows) in A-D.

**Fig 3:**
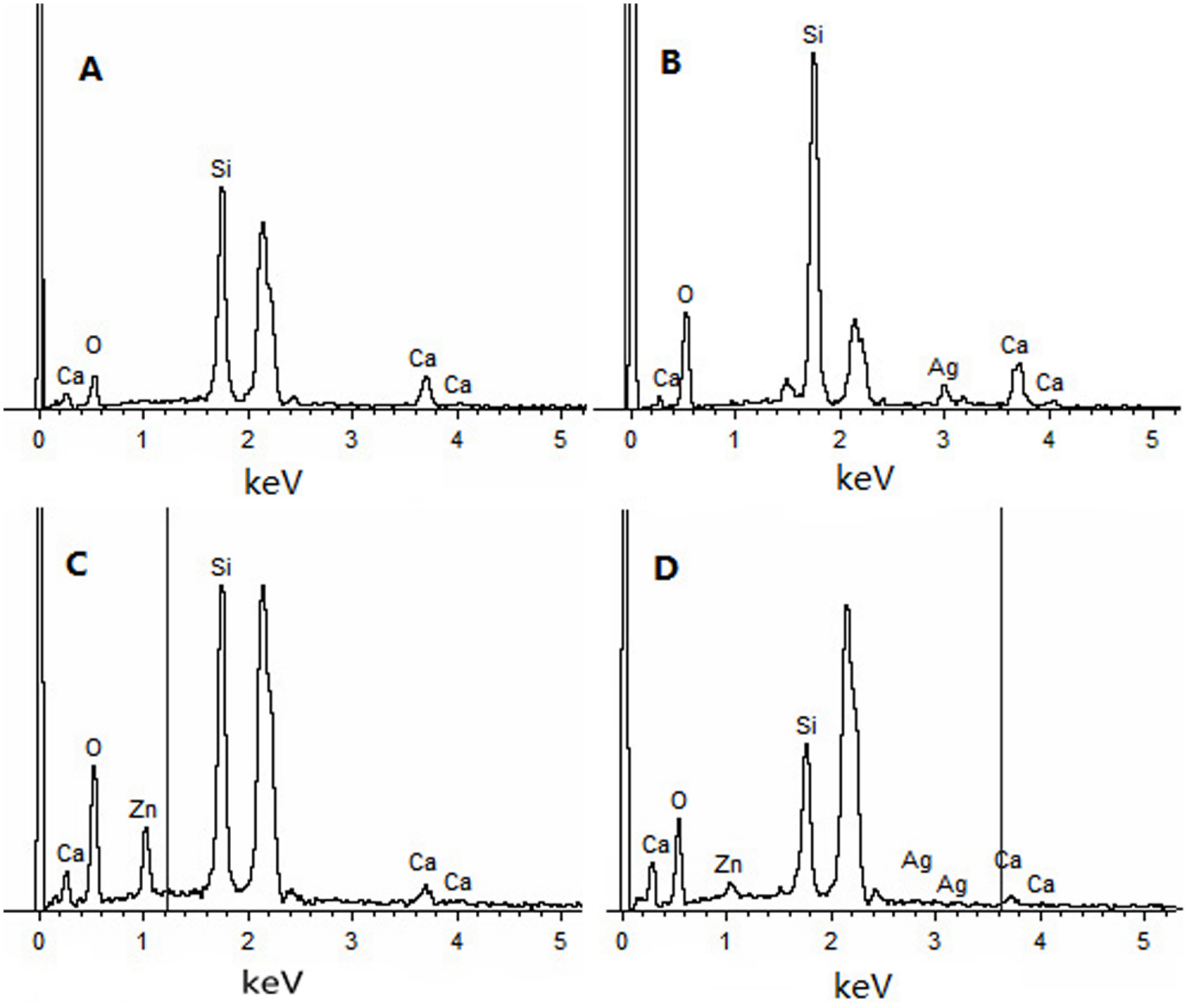
EDS of MCSNs, Ag-MCSNs, Zn-MCSNs, and Ag-Zn-MCSNs.

**Fig 4:**
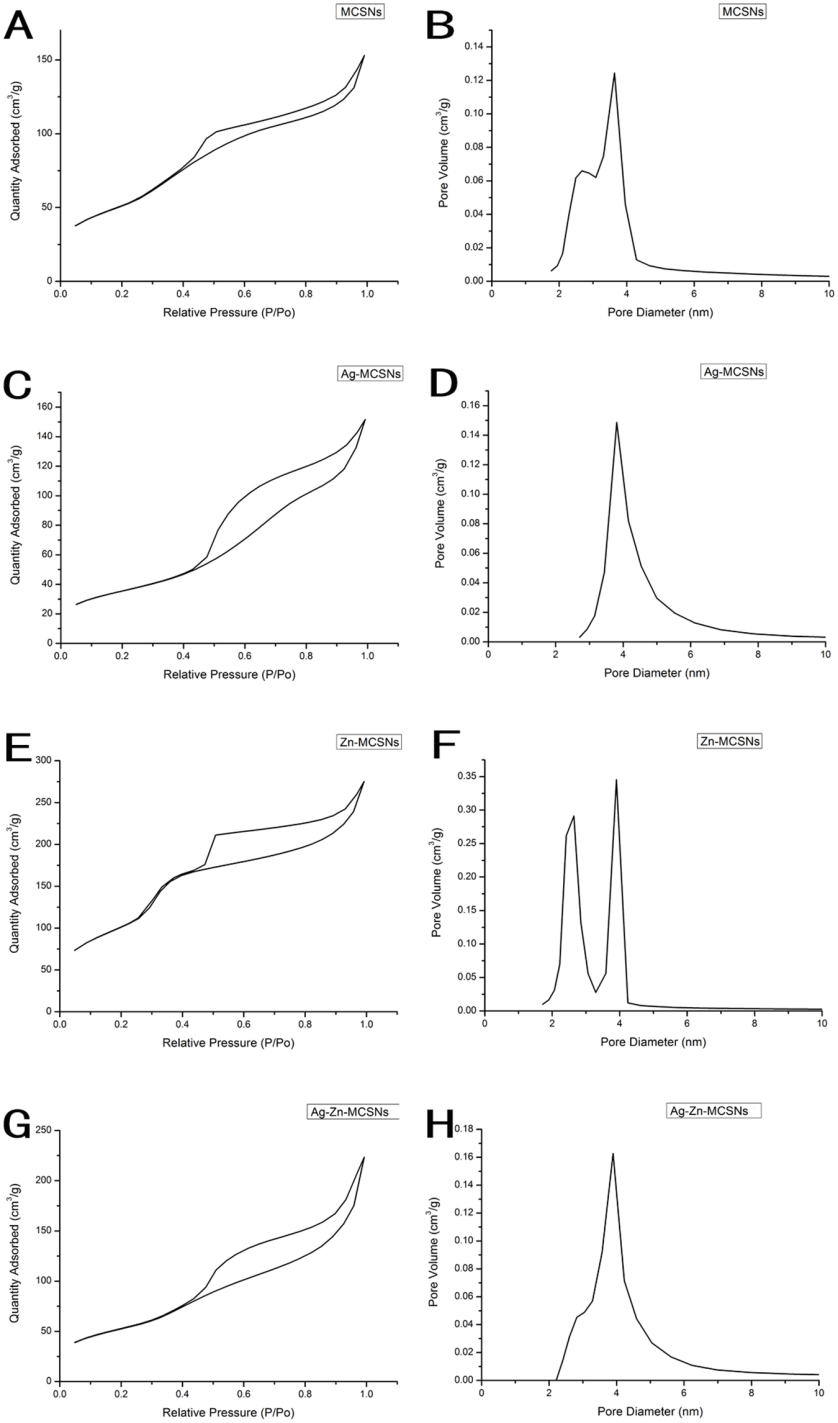
Nitrogen adsorption–desorption isotherm test and pore size distribution of MCSNs, Ag-MCSNs, Zn-MCSNs, and Ag-Zn-MCSNs.

The FE-SEM images showed that all the nanoparticles possessed spherical morphology with generally uniform size. The diameters of the nanoparticles were approximately 200–250 nm (Fig 1). TEM showed that the MCSNs, Ag-MCSNs, Zn-MCSNs and Ag-Zn-MCSNs had well-ordered nanopores and channel structures (Fig 2), and numerous black dots were trapped within the nanoparticles in the Ag-MCSNs and Ag-Zn-MCSNs (Fig 2B, 2D, 2b and 2d). The EDS analysis spectrum confirmed the presence of Ca and Si elements in all the nanoparticles and Ag and/or Zn in the Ag-MCSNs, Zn-MCSNs and Ag-Zn-MCSNs (Fig 3). The proportion of each element is shown in Table 2. Nitrogen adsorption-desorption isotherm of these nanoparticles revealed type IV isotherms with H1-type hysteresis loops and pore diameters mostly in the range of 5–7 nm (Fig 4). The diameter, specific surface area, pore volume, and average pore size of the nanoparticles are presented in Table 3.

**Table 2:** Quantitative EDS analysis of the element proportion in the nanoparticles (weight %).

Nanoparticles	Si	Ca	Ag	Zn
MCSNs	37.84	9.87	-	-
Ag-MCSNs	26.51	5.88	5.19	-
Zn-MCSNs	27.37	2.57	-	12.38
Ag-Zn-MCSNs	26.03	2.39	2.70	4.02

**Table 3:** Diameter(D), surface area (S_BET_), pore volume (V_P_), and mean pore size (D_P_) of the nanoparticles.

Materials	D(nm)	S_BET_(m^2^/g)	V_P_(cm³/g)	D_P_(nm)
MCSNs	209.84	189.78	0.24	4.99
Ag-MCSNs	249.30	125.96	0.23	7.45
Zn-MCSNs	249.53	385.36	0.43	4.42
Ag-Zn-MCSNs	252.88	188.55	0.35	7.33

### Ion release and pH change

**Fig 5:**
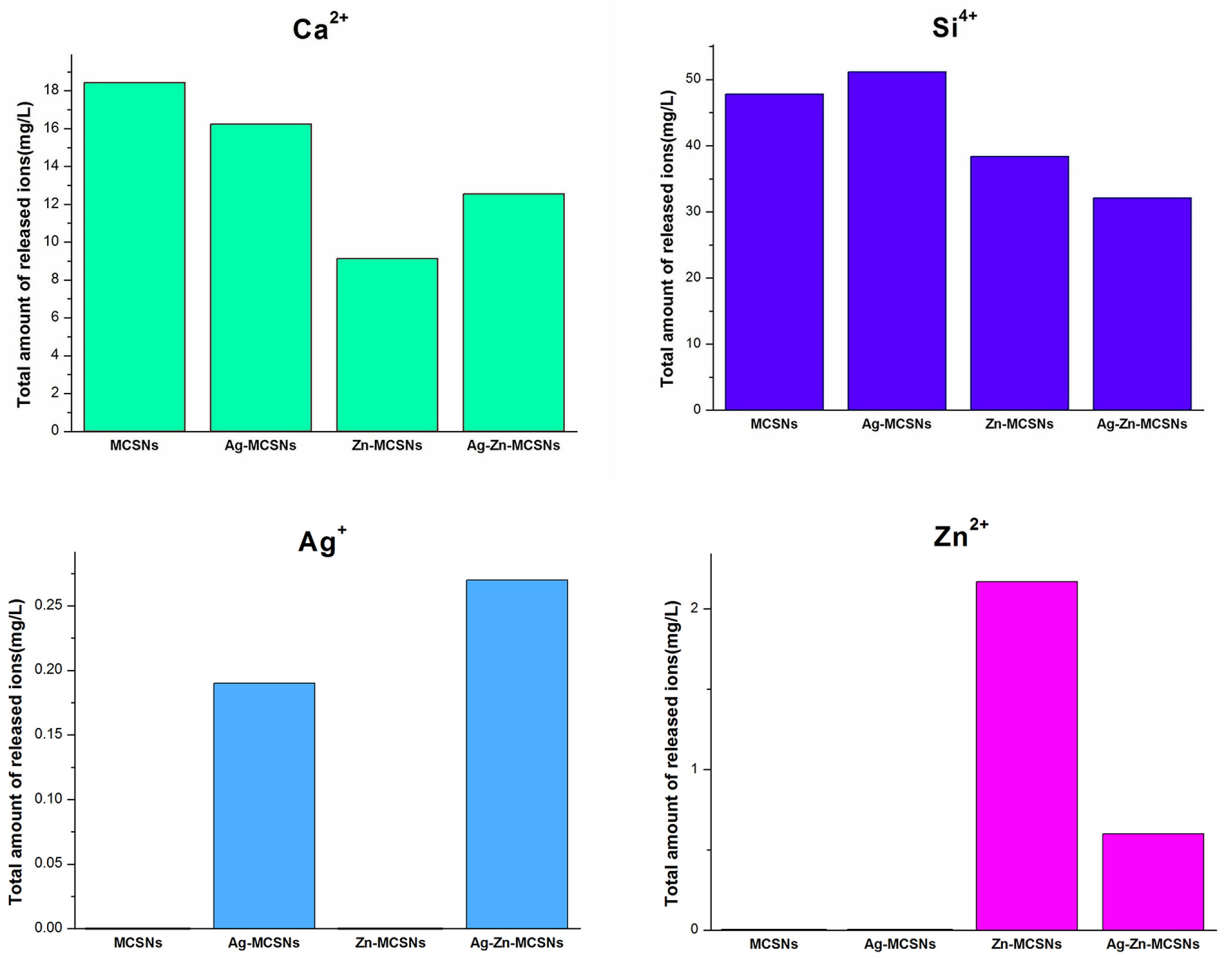
Ion release of MCSNs, Ag-MCSNs, Zn-MCSNs and Ag-Zn-MCSNs over time.

All the nanoparticles released Ca^2+^ and Si^4+^ in deionized water. The presence of metal ions changed the Ca^2+^ and Si^4+^ release rate of the nanoparticles. The Ag-Zn-MCSNs showed a faster release of Ag^+^ than the Ag-MCSNs and a slower release of Zn^2+^ than the Zn-MCSNs (Fig 5).

**Fig 6:**
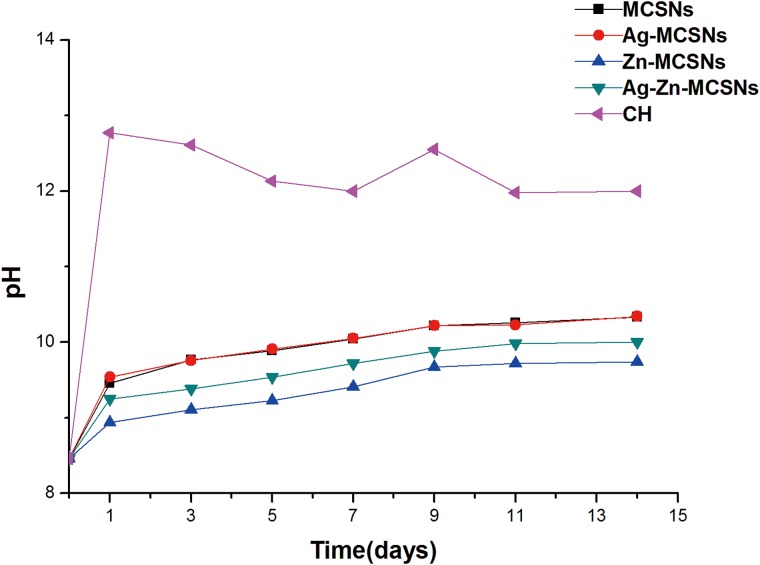
pH measurement of MCSNs, Ag-MCSNs, Zn-MCSNs, Ag-Zn-MCSNs and CH over time.

The pH of the MCSNs, Ag-MCSNs, Zn-MCSNs and Ag-Zn-MCSNs increased gradually over 14 days and was stable at 10. The addition of Ag did not affect the pH of the MCSNs significantly while the addition of Zn reduced the pH slightly. The pH of CH fluctuated slightly over time and stabilized at approximately 12 (Fig 6).

### Dentinal tubule infiltration

**Fig 7:**
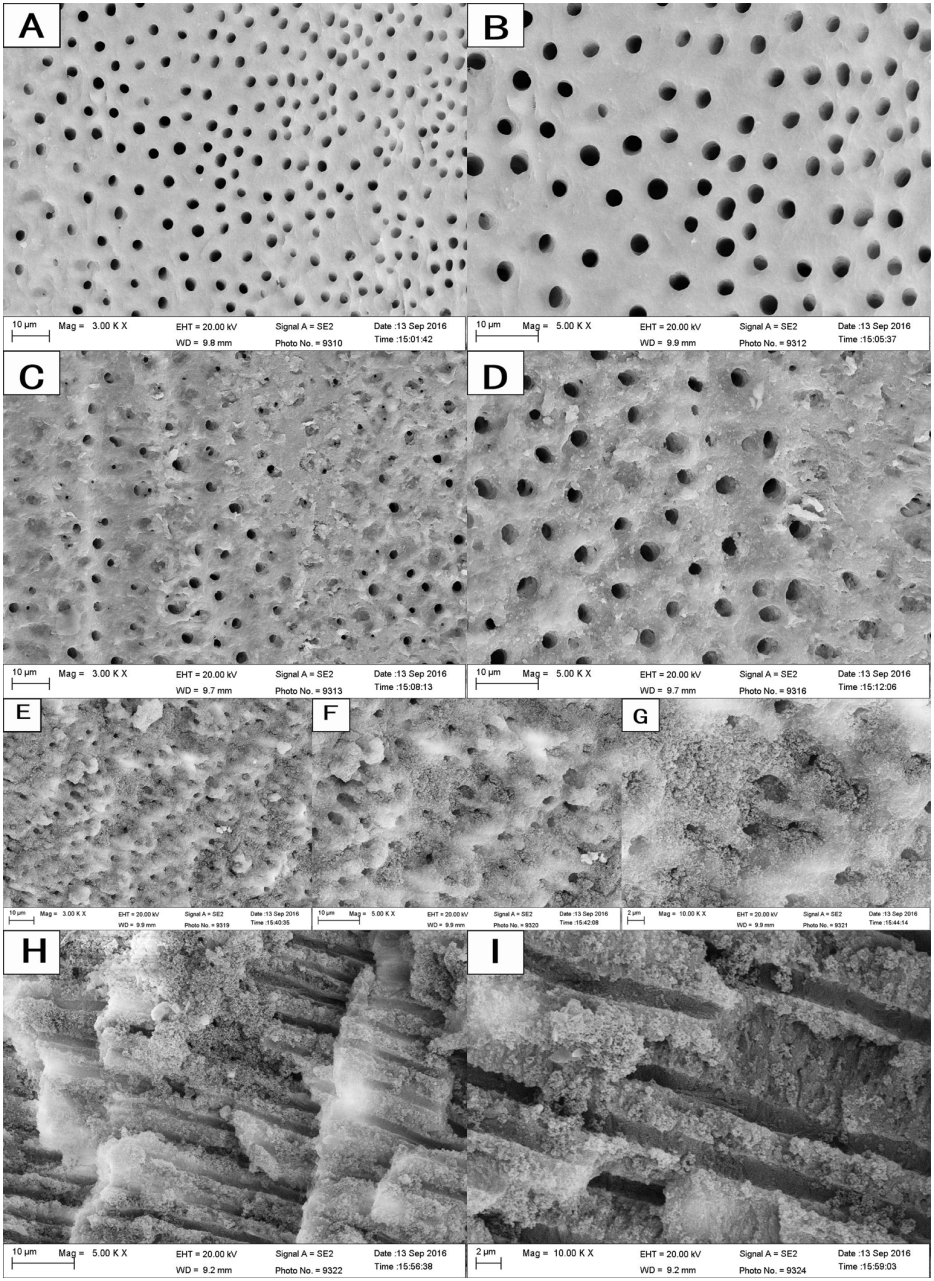
FE-SEM images of the nanoparticles covering the root canal wall and infiltrating the dentinal tubules.

**Fig 8:**
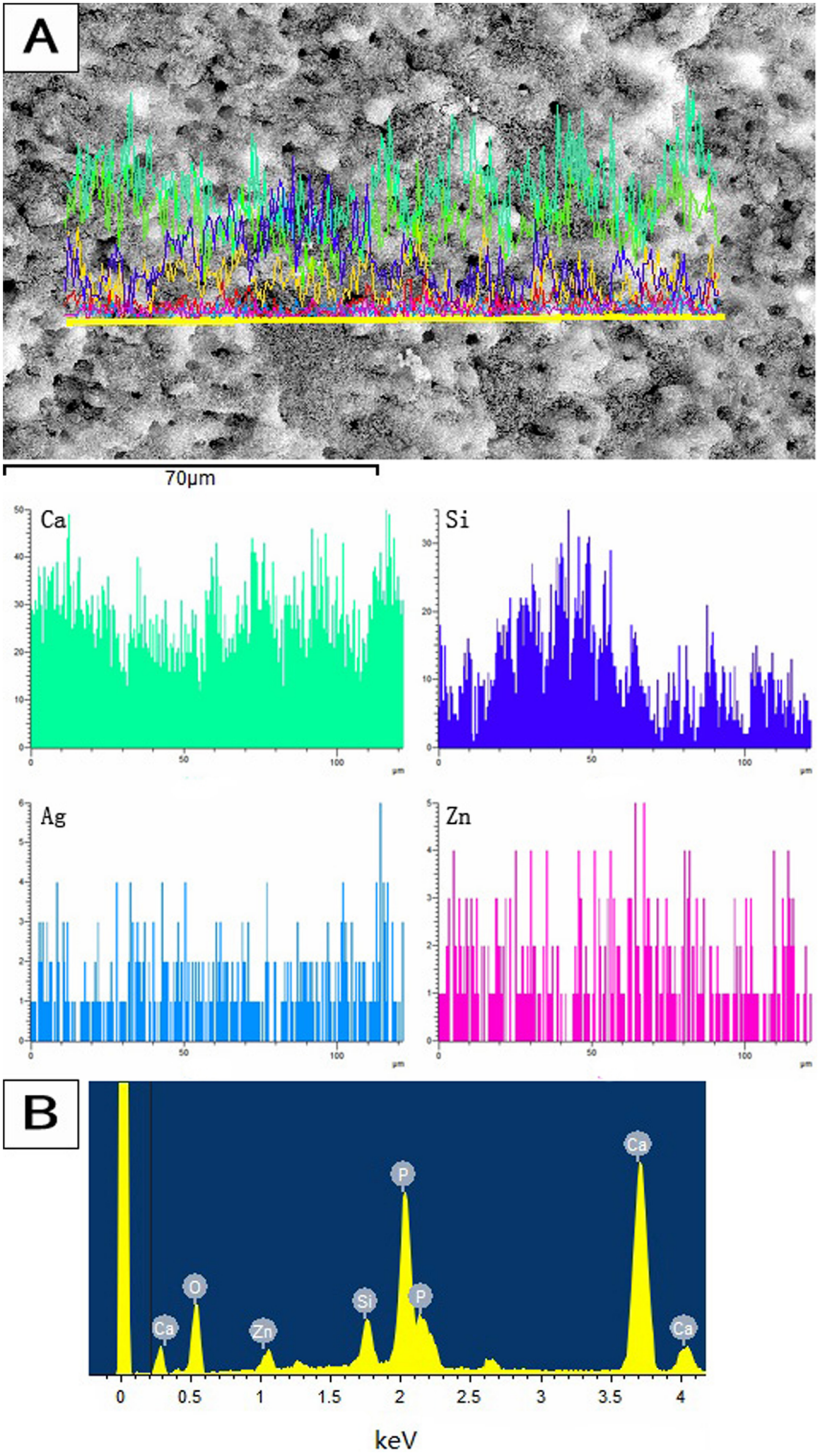
EDS results of the nanoparticles covering the root canal wall and infiltrating the dentinal tubules.

The FE-SEM images showed that some CH particles remained on the canal wall and covered the dentinal tubule orifices (Fig 7C and 7D). After passive ultrasonic treatment with Ag-Zn-MCSN suspension and canal rinsing, the canal wall and dentinal tubule orifices were covered with the nanoparticles (Fig 7E–7G). Numerous nanoparticles aggregated around the tubule orifices and infiltrated into the dentinal tubules (Fig 7H and 7I). EDS analyses confirmed the presence of Ag, Zn, Ca and Si in the nanoparticles covering the canal wall (Fig 8A) and the dentinal tubules (Fig 8B).

### Effects on the mechanical properties of root dentin

There was no difference in the flexural strength or the modulus of elasticity in the control group before and after soaking in saline. CH reduced the flexural strength of the dentin significantly (*P*<0.05), but had no significant effect on the modulus of elasticity (*P*>0.05). The effect of MCSNs, Ag-MCSNs, Zn-MCSNs, Ag-Zn-MCSNs on flexural strength and modulus of elasticity showed no statistical significance after 30 days compared with the control (*P*>0.05; Table 4).

**Table 4:** The drop or rise percentages of flexural strength and modulus of elasticity in CH, MCSNs, Ag-MCSNs, Zn-MCSNs, Ag-Zn-MCSNs.

Materials	flexural strength	modulus of elasticity
Saline	-0.68%	0.36%
CH	-9.41%*	2.4%
MCSNs	-2.02%	1.77%
Ag-MSCNs	-3.05%	1.1%
Zn-MCSNs	-2.43%	1.52%
Ag-Zn-MCSNs	-1.76%	2.41%

* Significantly different with control (*P*< .05).

## Discussion

MCSNs, Ag-MCSNs, Zn-MCSNs and Ag-Zn-MCSNs were successfully synthesized using a template method. These nanoparticles possess a typical mesoporous structure, and the nanosilver is distributed inside the mesoporous structure in the Ag-MCSNs and Ag-Zn-MCSNs. All the synthesized particles were on the nano scale, so they could be easily prepared as an injectable paste to fill the apical root canal of a tooth. Moreover, they possess high surface areas and pore volumes that can be used to deliver antibiotics with antibacterial effects and other bioactive molecules in their mesoporous structures to produce multifunctional biomaterials. In this study, all the nanoparticles could release Ca^2+^ and Si^4+^ produced a weak alkaline microenvironment and maintained a suitable pH value over time. In addition, the presence of Zn^2+^ promoted the release rate of Ag^+^. Ag and Zn have been found to have broad-spectrum antibacterial activity and have not induced resistance from target bacteria to date [11, 21]. Ag/Zn wireless electroceutical dressing markedly disrupted biofilm integrity in a setting when Ag dressing was ineffective [22]. Therefore, it is reasonably to speculate that the use of Zn^2+^ and Ag^+^ in nanoparticles may have good antibacterial effects in RCT.

Microorganisms play a fundamental role in the aetiology of pulp and periapical diseases. Infection of dentinal tubules within the intraradicular dentin has been reported to occur in 70%–80% of teeth with infection around the root apex. Facultative anaerobic bacteria such as *Enterococcus faecali*s are found in higher frequencies in failed root canal treatments. *Enterococcus faecali*s has a high tolerance for an alkaline environment and is able to penetrate the dentinal tubules to depths of 200 μm or more [7]. Bacterial invasion into dentinal tubules has been shown to be a major cause of posttreatment reinfection [23]. CH is the most widely used intracanal medicament, but there was no evidence that is had an increased antimicrobial effect when it was left for longer periods in the root canal because it has limited diffusion into the dentinal tubules and because of the buffering effect of dentin [5, 6, 24]. In this study, the Ag-Zn-MCSNs nanoparticles adhered well to the canal wall and infiltrated into the dentinal tubules even after canal rinsing. The nanoparticles could release Ag^+^ and Zn^2+^ in the dentinal tubules as antimicrobial agents, which may play an effective role in the disinfection of dentinal tubules.

The complete removal of CH from the root canal before filling is recommended because laboratory RCTs have revealed that CH remnants had negative effects [25]. Moreover, CH can weaken the flexural strength of the dentin and make the tooth more susceptible to fractures due to its strong alkaline nature, which can dissolve, denature or neutralize acidic organic components in the dentin tissue that act as bonding agents between hydroxylapatite crystals and collagenous fibrils [26, 27]. Marending *et al* [8] reported that CH could cause a 35% reduction in the mean flexural strength value of dentin after 10 days, White *et al* [28] discovered a 32% mean decrease in the strength of bovine dentin after exposure to CH for 5 weeks, and Sahebi *et al* [29] concluded that CH reduced the compressive strength of human root dentin by almost 15% after 30 days’ application. In addition, Grigorato *et al* [30] reported that the exposure of dentin to a saturated CH solution significantly reduced flexural strength but had no significant effect on the modulus of elasticity. In this study, CH showed a strong alkaline nature in deionized water and decreased the flexural strength of dentin significantly but had no significant effect on the modulus of elasticity. These findings were similar to previous experiments that studied the weakening effects of CH on the strength of root dentin and found that dentin exposed to CH for an extended period may present reduced flexural strength and low fracture resistance.

It was interesting to find that MCSNs, Ag-MCSNs, Zn-MCSNs and Ag-Zn-MCSNs had no significant negative effects on either the flexural strength or the modulus of elasticity of dentin. Studies have shown that calcium silicate-based bioactive glasses (BC) caused a 20% drop in the mean flexural strength of human root dentin after 10 days [8], while mineral trioxide aggregate (MTA) decreased the flexural strength of bovine dentin by approximately 28% after 30 days [31] and 33% after 5 weeks [28]. BC has a disinfecting capacity similar to that of CH because of the high pH and osmotic effects caused by the nonphysiological concentration of ions dissolved from the glass [32, 33]. MTA has an initial pH of 10.2 that increase rises to 12.5 three hours after mixing [34]. In this study, the pH of MCSNs, Ag-MCSNs, Zn-MCSNs and Ag-Zn-MCSNs in deionized water increased gradually over time but was stable at approximately 10. The absence of hydroxyl ions in aqueous solutions of these nanoparticles may explain why they had no significant negative effects on dentin. However, further studies are needed to conform the effects of these nanoparticles on the mechanical properties of human root dentin when they are used for a longer period of time.

Based on the findings in this study, these nanoparticles may be developed into a desirable intracanal disinfectant for the root canal as they were able to infiltrate the dentinal tubules and had no negative effects on the mechanical properties of dentin. Our previous studies indicated that Ag-MCSNs showed significant antibacterial effects[19], although it is not known whether Zn-MCSNs and Ag-Zn-MCSNs had better antibacterial properties than Ag-MCSNs. The biological stability or compatibility of these nanoparticles in cells, tissue or animals is also unclear. Further studies will be conducted to investigate these issues in the future.

## Conclusions

Within the limits of this study, it may be concluded that nanosilver and nanozinc can be successfully incorporated into MCSNs using a template method. The nanoparticles showed good characteristics, and had no negative effects on the mechanical properties of dentin. Ag-Zn-MCSNs adhered well to the root canal walls and infiltrated into the dentinal tubules, characteristics that could be developed into a new intracanal disinfectant for root canals.

## Supporting information

S1 Table Ions release of the nanoparticles. (DOC)

S2 Table The drop or rise percentages of flexural strength and modulus of elasticity. (DOC)
