# Supplementary material for: Effects of nanosilver and nanozinc incorporated mesoporous calcium-silicate nanoparticles on the mechanical properties of dentin
Source: PLoS One. 2017 Aug 7;12(8):e0182583. doi: 10.1371/journal.pone.0182583 (PMC5546636; doi:10.1371/journal.pone.0182583)
Supplement: S1 Table — (DOC) [file pone.0182583.s001.doc]

|  | **MCSNs** | **Ag-MCSNs** | **Zn-MCSNs** | **Ag-Zn-MCSNs** |
| --- | --- | --- | --- | --- |
| **Ca (mg/L)** | 18.43395 | 16.24175 | 9.12895 | 12.5467 |
| **Si (mg/L)** | 47.7905 | 51.13935 | 38.366 | 32.11755 |
| **Ag (mg/L)** | 0 | 0.1933 | 0 | 0.2717 |
| **Zn (mg/L)** | 0 | 0 | 2.1678 | 0.5961 |
