# Supplementary material for: Effects of nanosilver and nanozinc incorporated mesoporous calcium-silicate nanoparticles on the mechanical properties of dentin
Source: PLoS One. 2017 Aug 7;12(8):e0182583. doi: 10.1371/journal.pone.0182583 (PMC5546636; doi:10.1371/journal.pone.0182583)
Supplement: S2 Table — (DOC) [file pone.0182583.s002.doc]

**The drop or rise percentages of flexural strength**

| **Saline** | **CH** | **MCSNs** | **Ag- MCSNs** | **Zn- MCSNs** | **Ag-Zn- MCSNs** |
| --- | --- | --- | --- | --- | --- |
| -0.5587 | -0.3302 | -0.3354 | -0.0016 | -0.0747 | -0.1988 |
| 0.8701 | -0.4239 | 0.1976 | -0.1671 | -0.5482 | 0.0873 |
| -0.1433 | -0.1836 | 0.1056 | -0.2581 | 0.2114 | -0.1218 |
| 0.0607 | 0.0996 | 0.5327 | 0.0569 | -0.1412 | -0.1132 |
| -0.1181 | 0.0961 | 0.6487 | 0.0901 | -0.0315 | -0.0042 |
| -0.0137 | -0.4382 | 0.7640 | 0.1307 | -0.2020 | -0.2803 |
| -0.1435 | -0.0897 | -0.4449 | -0.0768 | -0.2503 | -0.0057 |
| -0.3183 | 0.1392 | -0.6630 | 0.8419 | 0.1618 | 0.2294 |
| -0.1850 | -0.3743 | 0.0345 | -0.0915 | 0.2180 | 0.1759 |
| 0.0920 | 0.0434 | 0.2844 | -0.0366 | -0.2216 | -0.3351 |
| 0.4889 | 0.8835 | 0.1627 | -0.1509 | 0.2405 | 0.0388 |
| -0.3663 | 0.1290 | 0.0032 | -0.2256 | 0.3420 | -0.0718 |
| -0.2069 | -0.3530 | -0.2495 | 0.3370 | 0.5755 | 0.1607 |
| -0.0479 | 0.2169 | -0.2999 | -0.4309 | -0.2359 | 0.2521 |
| 0.3924 | -0.1742 | -0.0505 | -0.5294 | -0.1341 | 0.0864 |
| 0.0621 | -0.3489 | -0.1590 | -0.0347 | -0.1884 | -0.1755 |
| 0.5274 | -0.4177 | -0.2000 | 0.6726 | -0.3206 | 0.1572 |
| -0.1999 | -0.0861 | -0.1893 | -0.0885 | -0.0510 | 0.0087 |
| 0.0591 | -0.5091 | -0.0359 | -0.3216 | -0.0447 | -0.2238 |
| 0.0318 | -0.0667 | -0.1777 | -0.2775 | 0.1866 |  |
| 0.0175 | 0.7142 | -0.3528 | 0.3015 | -0.0017 |  |
| -0.4503 | -0.0549 |  | -0.4112 |  |  |
|  | -0.2172 |  |  |  |  |
|  | -0.5126 |  |  |  |  |

**The drop or rise percentages of modulus of elasticity**

| **Saline** | **CH** | **MCSNs** | **Ag- MCSNs** | **Zn- MCSNs** | **Ag-Zn- MCSNs** |
| --- | --- | --- | --- | --- | --- |
| -0.1163 | 0.1343 | -0.1325 | -0.4733 | -0.4368 | -0.2648 |
| 0.0528 | -0.2976 | 0.2356 | -0.1313 | -0.4260 | 0.5808 |
| 0.1929 | 0.2854 | -0.2284 | -0.5217 | 2.2553 | 0.2236 |
| 0.3049 | -0.0898 | 0.5645 | 0.6672 | 0.4697 | -0.0120 |
| -0.2267 | 0.0343 | 0.0379 | 0.2817 | -0.1224 | -0.1009 |
| -0.2213 | -0.3570 | 0.2035 | 0.3772 | 0.0245 | 0.0210 |
| -0.0322 | 0.1838 | -0.2396 | -0.2065 | 0.0602 | -0.3008 |
| -0.2632 | -0.3054 | -0.4000 | 0.0558 | -0.0871 | 0.1066 |
| -0.2979 | 0.9830 | 0.5925 | -0.1770 | 0.0783 | 0.2345 |
| -0.2106 | -0.0399 | 0.1278 | 0.2504 | -0.6118 | -0.1497 |
| 0.1369 | 0.4405 | 0.1197 | 0.5988 | 0.0095 | -0.1714 |
| -0.0961 | -0.4122 | 0.5193 | -0.1772 | -0.0780 | 0.2755 |
| -0.2598 | -0.3013 | 0.2746 | -0.0381 | 0.0071 | 0.1454 |
| -0.2773 | 0.1381 | -0.1621 | -0.0112 | 0.1598 | -0.1192 |
| -0.1678 | 0.0652 | 0.0774 | 0.7088 | -0.8404 | 0.0886 |
| -0.1492 | 0.1456 | 0.1327 | 0.0955 | 0.0582 | -0.0490 |
| 0.1500 | 0.2524 | -0.5348 | -0.6047 | -0.3449 | -0.2342 |
| -0.0960 | -0.3728 | -0.1447 | -0.1189 | -0.1858 | 0.0947 |
| 0.4222 | -0.2810 | 0.2317 | -0.1947 | -0.0012 | 0.0887 |
| 0.7970 | -0.2850 | -0.3496 | 0.0022 | 0.1341 |  |
| 0.2810 | 0.6156 | -0.5532 | 0.0793 | 0.1973 |  |
| 0.1566 | 0.3437 |  | -0.2197 |  |  |
|  | -0.1248 |  |  |  |  |
|  | -0.1782 |  |  |  |  |
